# Validity of continuous glucose monitoring for categorizing glycemic responses to diet: implications for use in personalized nutrition

**DOI:** 10.1093/ajcn/nqac026

**Published:** 2022-02-04

**Authors:** Jordi Merino, Inbar Linenberg, Kate M Bermingham, Sajaysurya Ganesh, Elco Bakker, Linda M Delahanty, Andrew T Chan, Joan Capdevila Pujol, Jonathan Wolf, Haya Al Khatib, Paul W Franks, Tim D Spector, Jose M Ordovas, Sarah E Berry, Ana M Valdes

**Affiliations:** Diabetes Unit and Center for Genomic Medicine, Massachusetts General Hospital, Boston, MA, USA; Programs in Metabolism and Medical & Population Genetics, Broad Institute, Cambridge, MA, USA; Department of Medicine, Harvard Medical School, Boston, MA, USA; Zoe Ltd, London, United Kingdom; Department of Nutritional Sciences, King's College London, London, United Kingdom; Department of Twin Research and Genetic Epidemiology, King's College London, London, United Kingdom; Zoe Ltd, London, United Kingdom; Zoe Ltd, London, United Kingdom; Department of Medicine, Harvard Medical School, Boston, MA, USA; Diabetes Research Center, Massachusetts General Hospital, Boston, MA, USA; Clinical and Translational Epidemiology Unit, Massachusetts General Hospital and Harvard Medical School, Boston, MA, USA; Division of Gastroenterology, Massachusetts General Hospital and Harvard Medical School, Boston, MA, USA; Zoe Ltd, London, United Kingdom; Zoe Ltd, London, United Kingdom; Zoe Ltd, London, United Kingdom; Department of Clinical Sciences, Lund University, Malmö, Sweden; Department of Nutrition, Harvard TH Chan School of Public Health, Boston, MA, USA; Department of Twin Research and Genetic Epidemiology, King's College London, London, United Kingdom; Jean Mayer USDA Human Nutrition Research Center on Aging at Tufts University, Boston, MA, USA; IMDEA Food Institute, Campus of International Excellence (CEI) Autonomous University of Madrid + Higher Council for Scientific Research (UAM + CSIC), Madrid, Spain; Department of Nutritional Sciences, King's College London, London, United Kingdom; School of Medicine, University of Nottingham, Nottingham, United Kingdom; National Institute for Health Research Nottingham Biomedical Research Centre, Nottingham, United Kingdom

**Keywords:** continuous glucose monitoring, precision nutrition, meal responses, diet, glycemic variability

## Abstract

**Background:**

Continuous glucose monitor (CGM) devices enable characterization of individuals’ glycemic variation. However, there are concerns about their reliability for categorizing glycemic responses to foods that would limit their potential application in personalized nutrition recommendations.

**Objectives:**

We aimed to evaluate the concordance of 2 simultaneously worn CGM devices in measuring postprandial glycemic responses.

**Methods:**

Within ZOE PREDICT (Personalised Responses to Dietary Composition Trial) 1, 394 participants wore 2 CGM devices simultaneously [*n* = 360 participants with 2 Abbott Freestyle Libre Pro (FSL) devices; *n* = 34 participants with both FSL and Dexcom G6] for ≤14 d while consuming standardized (*n* = 4457) and ad libitum (*n* = 5738) meals. We examined the CV and correlation of the incremental area under the glucose curve at 2 h (glucose_iAUC0–2 h_). Within-subject meal ranking was assessed using Kendall τ rank correlation. Concordance between paired devices in time in range according to the American Diabetes Association cutoffs (TIR_ADA_) and glucose variability (glucose CV) was also investigated.

**Results:**

The CV of glucose_iAUC0–2 h_ for standardized meals was 3.7% (IQR: 1.7%–7.1%) for intrabrand device and 12.5% (IQR: 5.1%–24.8%) for interbrand device comparisons. Similar estimates were observed for ad libitum meals, with intrabrand and interbrand device CVs of glucose_iAUC0–2 h_ of 4.1% (IQR: 1.8%–7.1%) and 16.6% (IQR: 5.5%–30.7%), respectively. Kendall τ rank correlation showed glucose_iAUC0–2h_-derived meal rankings were agreeable between paired CGM devices (intrabrand: 0.9; IQR: 0.8–0.9; interbrand: 0.7; IQR: 0.5–0.8). Paired CGMs also showed strong concordance for TIR_ADA_ with a intrabrand device CV of 4.8% (IQR: 1.9%–9.8%) and an interbrand device CV of 3.2% (IQR: 1.1%–6.2%).

**Conclusions:**

Our data demonstrate strong concordance of CGM devices in monitoring glycemic responses and suggest their potential use in personalized nutrition.

This trial was registered at clinicaltrials.gov as NCT03479866.

## Introduction

Previous studies have reported high interindividual variability in postprandial glycemic responses to identical meals (1–4), stressing the need for more personalized nutritional approaches to reduce the unfavorable impact of glycemic excursions on obesity, diabetes, and related complications (5–8). By measuring interstitial glucose every 5–15 min, continuous glucose monitors (CGMs) better characterize individuals’ dynamic glycemic profiles in response to physiological and environmental stimuli than point-in-time glucose quantification approaches (9, 10). Furthermore, the dynamic assessment of glycemic variability (GV) enabled by CGMs offers insight into other features of postprandial glycemic responses besides 2-h incremental area under the glucose curve (glucose_iAUC0–2 h_) (11). Features including peak concentration, nadirs “below baseline,” time in range (TIR), and GV have been associated with pathophysiological conditions such as oxidative stress and inflammation (12–14) and physiological changes including hunger and energy intake (15). These findings highlight the utility of CGMs to expose the complexity and nuances in glycemic excursions in both healthy individuals and people with metabolic diseases.

With the increased demand for CGM use in healthy populations and the emergence of remote clinical testing and citizen science, questions have been raised regarding the reliability of CGMs in correctly ranking (categorizing) glycemic responses to foods and meals (16, 17). A recent study including 16 healthy adults who wore 2 CGM devices simultaneously reported highly discordant meal rankings between devices, suggesting that meal categorization is device-dependent (17). Although these results are important, because they call into question the use of CGM devices for precision nutrition applications, they rely on a single domiciled feeding study and warrant further replication.

Here, we leveraged data from the ZOE PREDICT (Personalised Responses to Dietary Composition Trial) 1 study including 394 healthy participants monitored with 2 CGM devices in parallel who consumed 4457 standardized meals and 5738 ad libitum meals during a 14-d period to investigate the repeatability of CGM devices in a free-living setting.

## Methods

### Study design and population

ZOE PREDICT 1 (NCT03479866) was a single-arm, multicenter intervention study to investigate variations in postprandial responses to standardized meals and ad libitum free-living foods based on individuals’ characteristics, as described elsewhere (4). In brief, ZOE PREDICT 1 enrolled 1002 healthy individuals aged 18–65 y with no recent diagnosis of metabolic, inflammatory, or mental health diseases, dietary restrictions, and not taking medications that could influence metabolism. We excluded individuals with type 1 diabetes, those taking antidiabetic medications, and those with a capillary glucose concentration >12 mmol/L based on fingertip glucose measurements. Participants were enrolled in a 14-d intervention consisting of test meal challenges of various nutritional content (**Supplemental Table 1**), including 1 full day of clinical measurements with a controlled test meal challenge at baseline. Participants wore digital devices including CGM sensors and recorded all foods and drinks consumed via a mobile phone application specially designed for the study. Primary outcomes are reported elsewhere (4, 18) and include gut microbiome profile, blood lipids and glucose, sleep, physical activity, and hunger and appetite assessment. Data for the secondary outcome of CGM interdevice concordance in a subgroup only are reported in this article. Participants were invited to take part in this secondary analysis based on their period of enrollment into the study (October 2019–April 2021) and no additional recruitment criteria were applied to this subgroup.

Ethical approval for the study was obtained in the United Kingdom from the Research Ethics Committee and Integrated Research Application System (IRAS 236407) and in the United States from the Partners Healthcare institutional review board (IRB 2018P002078). The trial was conducted in accordance with the Declaration of Helsinki and Good Clinical Practice.

### CGM devices

Participants’ glucose was measured continuously throughout the study period using digital CGM devices. As part of this secondary analysis, a total of 394 ZOE PREDICT 1 participants were monitored with 2 CGM devices worn in parallel during the study period. Among them, 360 individuals were monitored with the Abbott Freestyle Libre Pro CGM (FSL; referred to as Device A), and 34 participants simultaneously wore the FSL and the Dexcom G6 CGM (DEX; referred to as Device B).

Monitors were fitted by trained nurses on the upper arms, 1/side, at participants’ baseline visit and were covered with Opsite Flexifix adhesive film (Smith & Nephew Medical Ltd) for improved durability. The rationale to place the DEX in the upper arm instead of the lower abdomen, as suggested per manufacturer specifications, was to reduce the burden to participants caused by monitoring at 2 separate sensor locations, without compromising sensor accuracy (19). The glucose concentration in the interstitial compartment in which CGM devices operate mirrors that of the intravascular compartment within minutes, thus making this a useful glycemic metric for both locations (20). FSL devices were worn for the entire study duration, whereas DEX devices have a shorter recording limit and were therefore worn until day 12 (while ensuring all participants completed all standardized test meal days). Subjects were blinded to CGM glucose readings.

Once removed, CGM devices were mailed back to study staff. The data for each monitor were downloaded at the end of each study period. Per manufacturer instructions and a previous study of longitudinal accuracy, only those CGM data points collected from 12 h onwards after activating the device were considered (21, 22).

### Standardized and ad libitum meals

Participants’ postprandial glycemia was measured in response to both standardized and ad libitum meals. **Supplemental Tables** 1 and **2** detail nutritional characteristics of standardized meals consumed during the study intervention and the consumption protocol. Briefly, standardized test meals consisted of a metabolic challenge breakfast and lunch meal containing 1390 kcal combined; a range of 500-kcal meals delivered in the form of muffins, milkshakes, and energy bars; and finally a 300-kcal oral-glucose-tolerance test (OGTT), consumed at home after overnight fasts. The meals varied in their nutritional compositions (ranges: 28–95 g carbohydrate, 0–53 g fat, and 0–41 g protein), and were consumed as breakfast and lunch of days 2–3 and as breakfast alone during days 4–12. Standardized meals were consumed either singularly or in duplicate, and the meal order was block randomized (23).

In addition to standardized meals, participants consumed an ad libitum diet during the at-home study period. Participants were trained to accurately weigh and record ad libitum dietary intake using photographs, product barcodes, product-specific portion sizes, and digital scales over the entire 14-d study period (4). Data logged into the study app were uploaded onto a digital dashboard in real time and assessed for logging accuracy and study compliance by study staff (**Supplemental Methods 1**). Any uncertainties were clarified actively with the participant through the app's messaging system or via phone while on the study.

All meals were reviewed by study staff in real time to assess the accuracy of logged meals and compliance in consuming the standardized test meals. As part of our quality control assessment, we excluded meals containing <70 kcal and where the meal quantity consumed was <15 g, and meals that had incomplete time points.

### Outcome measures

The primary outcome of this secondary study was repeatability of CGM measures for glucose_iAUC0–2 h_, in response to standardized and ad libitum free-living meals. Repeatability was assessed using the intrabrand device and interbrand device CVs of glucose_iAUC0–2 h_. These analyses were restricted to participants with ≥2 meals passing internal quality checks (*n* = 359 intrabrand device group; *n* = 34 interbrand device group) (**Supplemental Figure 1**). To avoid overlap between ≥2 glucose responses to food items consumed at temporal proximity, glucose_iAUC0–2 h_ was derived only from those foods and meals consumed within 30 min of each other as noted by participants’ real-time digital diet logs, or standardized meals followed by the prescribed 3- to 4-h fasting period. We also investigated the correlation of meal ranking within participants derived from paired glucose_iAUC0–2 h_ readings and the concordance of between-person meal ranking for glucose_iAUC0–2 h_.

Secondary outcomes included time in range according to the American Diabetes Association cutoffs (TIR_ADA_) (24), as well as an alternate cutoff designed for individuals without diabetes [time in range according to nondiabetic adjusted cutoffs (TIR_ND_)]. We also considered glucose variability (glucose CV) calculated by dividing glucose SD by mean glucose.

### Statistical analysis

We summarized differences in clinical, demographic, and biochemical characteristics between individuals wearing 2 intrabrand or 2 interbrand devices using the Kruskal–Wallis test for continuous measurements or the chi-square test for categorical variables. We also assessed differences in baseline characteristics between participants wearing a double-device or single-device from the original PREDICT 1 cohort.

The mean glucose_iAUC0–2 h_ was calculated using the trapezoid rule with respect to the baseline glucose measurement, divided by the duration of the postprandial measurements. The mean glucose_iAUC0–2 h_ for standardized meals and high-carbohydrate ad libitum meals with >25 g carbohydrate was log and square root transformed, respectively, and normal distribution was tested using the Shapiro–Wilk test. The correlation between each pair of mean glucose_iAUC0–2 h_ readings derived from parallel-worn devices was analyzed by Pearson's correlation (if normally distributed) and Spearman's correlation (if not normally distributed), for both intrabrand and interbrand device comparisons. Bland–Altman analysis was done on the log-transformed glucose_iAUC0-2 h_ values from interbrand devices derived from all meal types as well as high-carbohydrate meals only to assess if meal carbohydrate content and magnitude of glucose_iAUC0–2 h_ biased device agreement.

Concordance between meal ranking of paired devices was investigated using the CV for each pair of readings of glucose_iAUC0–2 h_. A Kendall τ rank correlation was used to measure the agreement between paired CGM devices in ranking of meals, based on their glucose_iAUC0–2 h_ measures on a per-participant basis. Further analysis was done on between-person meal ranking, where individuals’ meal glucose_iAUC0–2 h_ measures were averaged per device to obtain a person rank relative to the rest of the study population, with 1 rank/device. The concordance in an individual's 2 rankings was analyzed between the top and bottom quintiles of ranks to calculate a percentage agreement. Because standardized meals were consumed in duplicate, intraindividual concordance between meal rankings for the same meal (OGTT) consumed on different days was investigated using the CV of the glucose_iAUC0–2 h_.

For analysis of time in range and glucose CV, we excluded participants with incomplete days and CGM malfunction (>25 readings at monitor baseline per day or >10% missing reads per day) from GV analysis (*n* = 5 wearing device A only; *n* = 1 wearing devices A and B), yielding a total of 388 participants (*n* = 355 intrabrand device group; *n* = 33 interbrand device group) (Supplemental Figure 1). In participants wearing the intrabrand and interbrand devices, the monitoring period ranged from 2 to 12 d and 2 to 9 d, respectively. The correlation between time in range readings derived from parallel-worn devices, as well as glucose CV, was analyzed by Pearson's correlation (if normally distributed) and Spearman's correlation (if not normally distributed), for both intrabrand and interbrand device comparisons.

Owing to differences in BMI between both groups, we conducted a sensitivity analysis after selecting a BMI-matched subcohort for intrabrand device participants that mirrored the interbrand device BMI characteristics. Then, we tested the correlation coefficient of glucose_iAUC0–2 h_ after a set meal between the 2 groups.

Two-sided *P* values < 0.05 were considered statistically significant for main analyses. Analyses were performed using R version 3.4.2 (R Core Team), Python version 3 (CreateSpace), and GraphPad Prism version 9.1.1 (GraphPad Software, Inc.).

## Results

### Concordance in glycemic responses

**Table 1** presents baseline demographic, anthropometric, and biochemical characteristics for the study participants. Participants in the intrabrand device group were more likely than participants in the interbrand group to be male and have higher BMI and cholesterol concentrations. Similar differences were observed between participants wearing duplicate CGMs and those wearing a single CGM (from the full ZOE PREDICT 1 UK cohort) (**Supplemental Table 3**). There were no significant differences in the glucose measures (glucose peak, rise, or baseline) between devices for the intrabrand or interbrand groups (**Supplemental Table 4**).

**TABLE 1:** Characteristics of PREDICT 1 study participants who wore two CGM devices simultaneously

Metric	Device A only (*n* = 360)	Devices A and B (*n* = 34)	*P* value
Demographic			
Sex, % female	69.7	44.1	0.000
Age, y	45.8 ± 9.8	43.6 ± 11.5	0.231
Anthropometry			
Weight, kg	73.8 ± 14.7	71.1 ± 14.6	0.410
Height, cm	169.0 ± 9.1	172.2 ± 11.0	0.023
BMI, kg/m^2^	25.8 ± 4.8	23.9 ± 3.7	0.018
Waist circumference, cm	84.9 ± 12.3	83.2 ± 10.5	0.450
Waist:hip ratio	0.84 ± 0.1	0.87 ± 0.1	0.117
Systolic BP, mm Hg	125.8 ± 14.2	125.7 ± 12.4	0.908
Diastolic BP, mm Hg	77.0 ± 10.3	74.8 ± 10.3	0.292
Biochemistry			
Triglyceride, mg/dL	1.1 ± 0.6	1.1 ± 0.6	0.780
Cholesterol, mmol/L	4.8 ± 0.9	4.5 ± 0.8	0.039
LDL cholesterol, mmol/L	3.2 ± 0.9	2.9 ± 0.8	0.032
HDL cholesterol, mmol/L	1.7 ± 0.4	1.5 ± 0.5	0.113
Glucose, mmol/L	4.9 ± 0.5	4.9 ± 0.4	0.462
Glucose iAUC, mmol × L^−1^ × min	6937.4 ± 2431.1	6940.9 ± 1860.6	0.630
HbA1c, %	5.4 ± 0.3	5.4 ± 0.3	0.430
Insulin, mIU/L	5.8 ± 3.9	6.0 ± 3.2	0.467
C-peptide, ug/L	1.1 ± 0.5	1.2 ± 0.6	0.306

**FIGURE 1:**
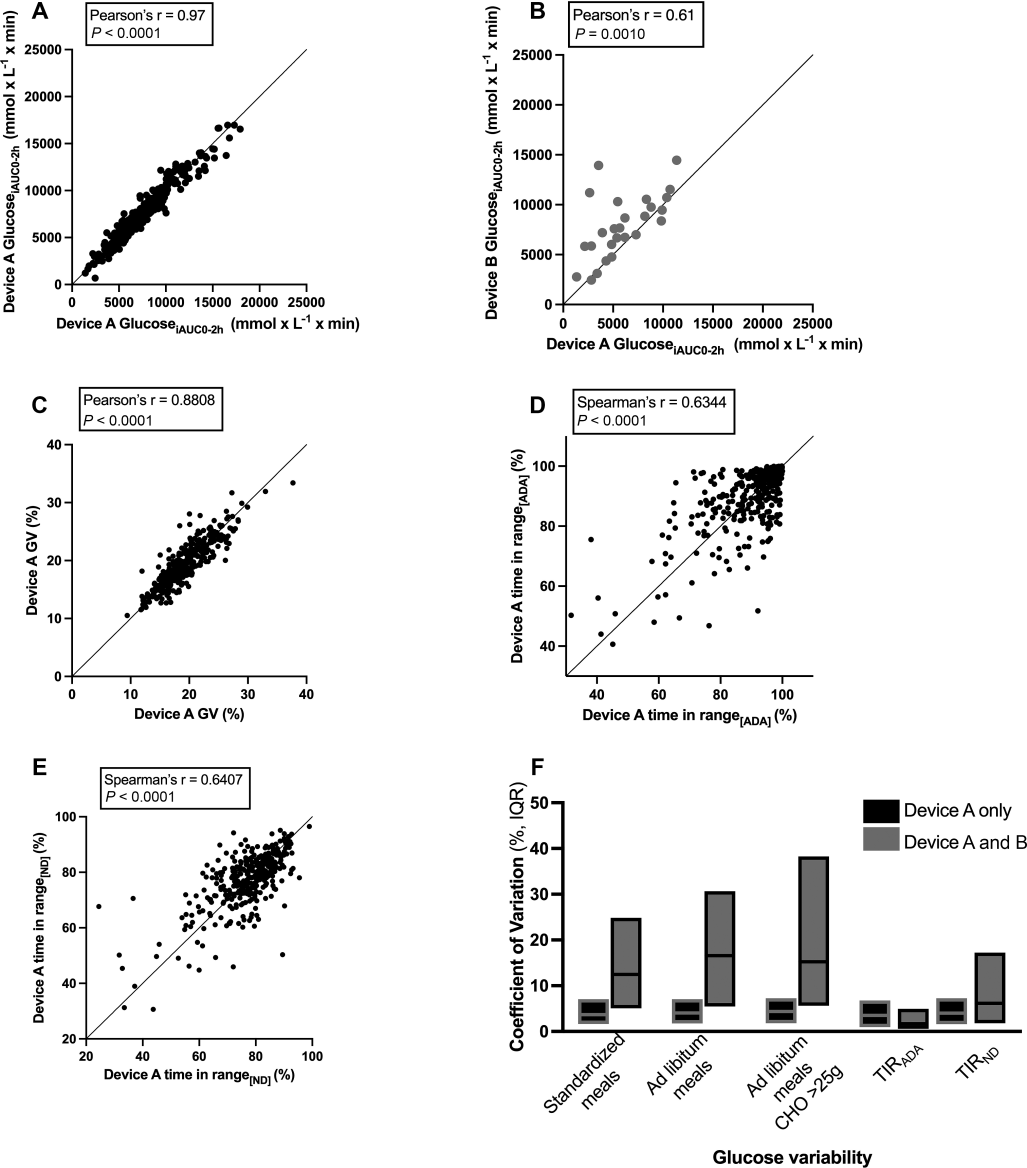
Correlation and concordance of glucose variability obtained from 2 CGM devices worn in parallel. (A, B) Pearson's correlation of glucose_iAUC0–2 h_ readings in response to ad libitum meals with high carbohydrate content (>25 g CHO), obtained from 2 (A) intrabrand (*n* = 338) and (B) interbrand (*n* = 26) CGMs. (C) Pearson's correlation of short-term GV in the form of glucose CV, from 2 intrabrand CGMs (*n* = 342). (D) Pearson's correlation of TIR_ADA_ from 2 intrabrand CGMs (no values in data set <40%, *n* = 342). (E) Pearson's correlation of TIR_ND_ from 2 intrabrand CGMs (no values in data set <20%, *n* = 342). (F) CV of glucose_iAUC0–2 h_ for standardized meals (*n* = 359 intrabrand pairs; *n* = 34 interbrand pairs), for ad libitum meals (*n* = 351 intrabrand pairs; *n* = 30 interbrand pairs), and for meals containing >25 g CHO (*n* = 338 intrabrand pairs; *n* = 26 interbrand pairs), as well as CVs for TIR_ND_ and TIR_ADA_ for paired intrabrand (*n* = 355) and interbrand (*n* = 33) CGMs. (A–E) Lines of *x* = *y* identity are presented. CGM, continuous glucose monitor; CHO, carbohydrate; glucose_iAUC0–2 h_, incremental area under the glucose curve between 0 and 2 h; GV, glycemic variability; TIR_ADA_, time in range according to American Diabetes Association cutoffs; TIR_ND_, time in range according to nondiabetic adjusted cutoffs.

The CV of glucose_iAUC0–2 h_ after standardized meals was 3.7% (IQR: 1.7%–7.1%) for intrabrand device and 12.5% (IQR: 5.1%–24.8%) for interbrand device comparisons (**Figure 1**). Ad libitum meals (carbohydrate median: 35 g; IQR: 20–57 g) showed slightly higher CVs of 4.1% (IQR: 1.8%–7.1%) for intrabrand device and 16.6% (IQR: 5.5%–30.7%) for interbrand device comparisons. When stratifying by carbohydrate content (> or <25 g), ad libitum meals containing >25 g carbohydrate (median: 50 g; IQR: 36–69 g) resulted in smaller CVs for both intrabrand (4.4%; IQR: 1.8%–7.3%) and interbrand device (15.2%; IQR: 5.6%–38.3%) comparisons than did ad libitum meals with all carbohydrate contents. Because meals were consumed in duplicate, the intraindividual CV of glucose_iAUC0–2 h_ after the same meal on different days was 29.5% (IQR: 12.7%–39.2%) for intrabrand device and 30.9% (IQR: 13.5%–38.8%) for interbrand device comparisons.

The correlation coefficient of glucose_iAUC0–2 h_ after all meal types combined was 0.97 (95% CI: 0.96, 0.98) for intrabrand device and 0.56 (95% CI: 0.28, 0.76) for interbrand device comparisons (**Supplemental Figure 2**). When studying ad libitum meals with high carbohydrate content (>25 g carbohydrate), paired CGMs again showed strong agreement between intrabrand devices (*r^2^* = 0.97; 95% CI: 0.96, 0.98) as well as between different brands (*r^2^* = 0.61; 95% CI: 0.29, 0.81) (Figure 1). Bland–Altman analysis demonstrated that discordance between interbrand devices was not biased by the carbohydrate content of meals or the magnitude of glucose_iAUC0–2 h_ (**Supplemental Figure 3**). In a sensitivity analysis conducted in a BMI-matched cohort to investigate if differences in BMI biased our results, we found that the correlation coefficient of glucose_iAUC0–2 h_ after a set meal was similar to the main analysis (*r^2^* = 0.97; 95% CI: 0.97, 0.98).

### Concordance in meal ranking

**FIGURE 2:**
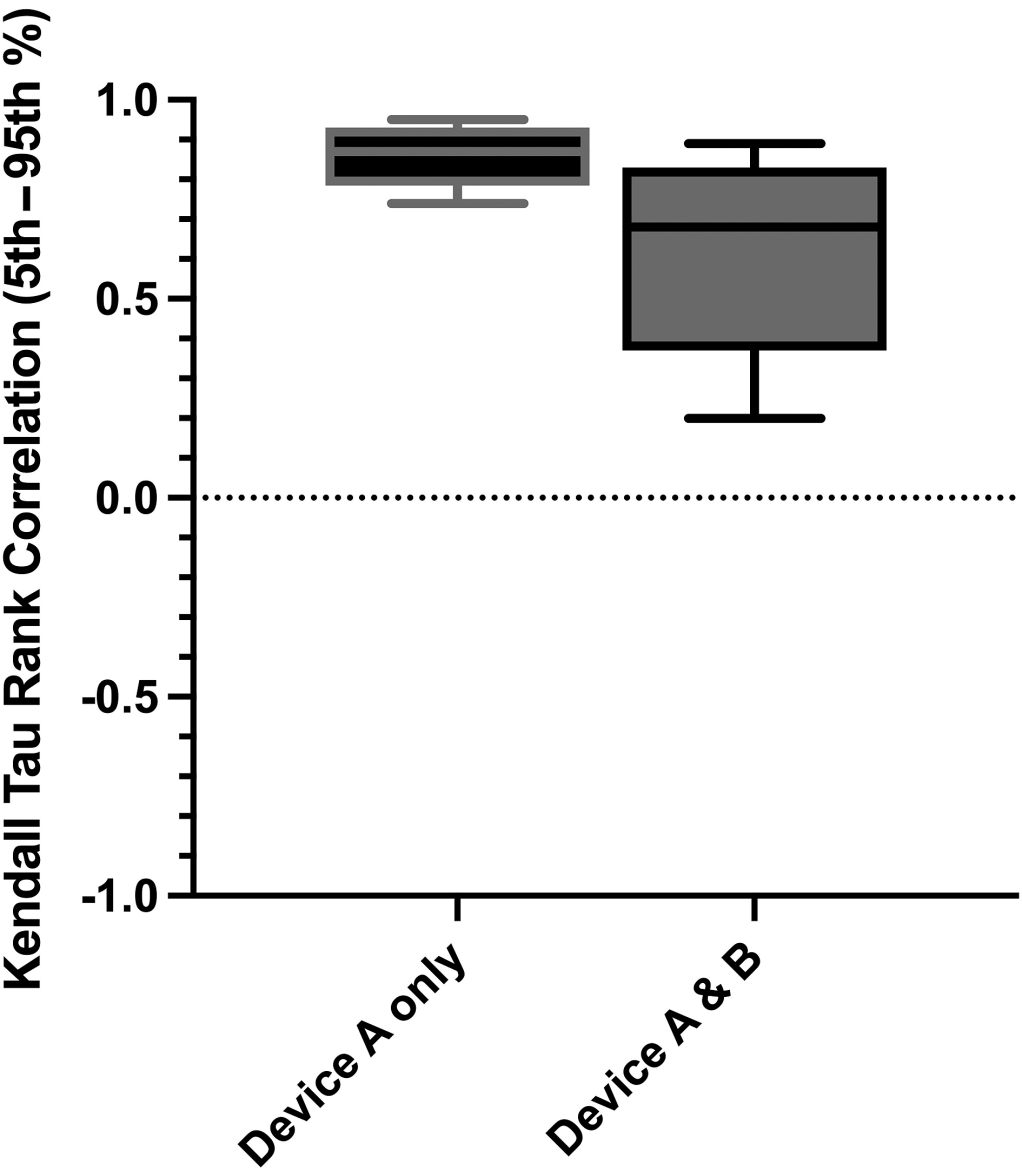
Kendall τ rank correlation of incremental area under the glucose curve between 0 and 2 h obtained from paired intrabrand (*n* = 359 subjects, 4406 meals) and interbrand (*n* = 34 subjects, 356 meals) continuous glucose monitors. Top and bottom barriers of boxes represent the interquartile range; the central line represents the median; the top and bottom brackets represent the maximum and minimum respectively.

Meal rankings for the glucose_iAUC0–2 h_ were concordant between paired CGM devices, with a mean Kendall τ rank correlation coefficient of 0.87 (IQR: 0.83–0.91) for intrabrand device and 0.68 (IQR: 0.54–0.77) for interbrand device comparisons (**Figure 2**). In addition, we investigated how likely a person was to be ranked in the top quintile of responders for a given ad libitum meal with 1 CGM and the bottom quintile with its paired CGM. We showed that the likelihood of misclassifying meals was small, with an intrabrand CGM discordance between the top- and bottom-ranked meals of 5% and interbrand discordance of 19%.

We next investigated the intrabrand device correlation for TIR_ADA_, TIR_ND_, and glucose CV. For these analyses we excluded 6 participants with incomplete days and CGM malfunction. Excluded participants were older and had higher mean BMI and cholesterol concentrations than those included in the main analysis (Supplemental Table 3). The population mean ± SD glucose variability as represented by glucose CV, TIR_ND_, and TIR_ADA_ was 19.4% ± 3.9%, 77.2% ± 10.5%, and 89.8% ± 11.4% in participants wearing device A only, respectively, and 18.2% ± 3.4%, 73.8% ± 13.0%, and 95.7% ± 7.2% in those wearing devices A and B, respectively. The correlation coefficient for glucose CV, TIR_ADA_, and TIR_ND_ was 0.88 (95% CI: 0.85, 0.90), 0.63 (95% CI: 0.55, 0.69), and 0.64 (95% CI: 0.57, 0.70), in participants wearing device A only, respectively (Figure 1) and 0.77 (0.59, 0.88), 0.34 (−0.01, 0.62), and 0.48 (0.15, 0.72) in those wearing devices A and B, respectively (Supplemental Figure 2). TIR_ADA_ and TIR_ND_ showed relatively low variability between parallel-worn devices, with a CV of 4.78% (IQR: 1.89%–9.77%) and 4.57% (2.24%–8.74%) for intrabrand devices, respectively, and a CV of 3.24% (1.08%–6.23%) and 7.47% (2.70%–18.7%) for interbrand device comparison, respectively.

## Discussion

In this study we provide quantitative data on the concordance of paired CGM sensors in monitoring glycemic responses. We report strong intrabrand agreement between paired devices in measuring glucose meal incremental AUC and glucose change metrics. We also show that the likelihood of meal-response misclassification between intrabrand devices is low. We report that agreement between sensors of different brands is lower than intrabrand agreement, although it is higher than has been reported elsewhere (17). Taken together, our findings highlight the potential application of CGMs for monitoring glycemic responses to foods and meals and their potential application for personalized nutrition recommendations in healthy populations.

Our analysis of daily glycemic change shows high sensor agreement relative to the acute measure of glucose_iAUC0–2 h_. The low CV values reported for all glycemic metrics here demonstrate the efficacy of CGMs in capturing different features of an individual's glycemic response to food and overall free-living dietary patterns. The considerable, albeit slightly lower, agreement between interbrand devices suggests that meal categorization is not only device-independent but also not biased by brand. Short-term glycemic change has been shown to be sensitive to dietary modifications in small studies focused on individuals with type 1 and type 2 diabetes. Our study is significant in that we report measures of concordance for time in range and glucose CV in a large population of generally healthy participants without diabetes.

Our findings are consistent with a clinical study (20) and a position statement from worldwide diabetes associations (25), showing that the mean absolute relative difference (MARD), a metric often used to assess CGM accuracy under different physiological or experimental conditions (26), is similar across new-generation CGM systems and ranges from 10% to 20%. However, we note that the data presented here do not replicate the recent study by Howard et al. (17), which reported high interbrand device discordance in meal rankings for incremental glycemic responses. The observed mean fraction of missing glycemic reduction determined by discordant CGM devices of ∼50% was much larger than that reported in the present study.

Several differences in study design and execution between the current study and that by Howard et al. may underlie the dissimilarity in results. Our study included a larger population size and tested the validity of intrabrand as well as interbrand CGM device concordance. In addition, we assessed glycemic responses after a mixture of standardized test meals, all ad libitum (regardless of carbohydrate content) meals, and ad libitum high-carbohydrate meals (>25 g). This distinction is critical in assessing the development of personalized nutrition guidelines that are tailored to an individual's glycemic response as measured by CGMs (1, 4). Furthermore, it is essential to note the nature of human nutritional studies, where it is common to use 1 single brand and version of a study device rather than a multitude. In this context, it can be appreciated that population studies accruing their data through a single device brand, as is common practice in the field, will result in improved device concordance, as we have shown here.

Potential reasons for the higher discordance in meal rankings reported by Howard et al. may also involve their inclusion of individuals from a domiciled feeding study randomly assigned to ad libitum ultra-processed or unprocessed diet (17) that resulted in a mean caloric intake > 3500 kcal in both groups. Excess caloric intake is likely to influence sensor precision, because it has been shown that CGM performs poorly at the very top or very bottom of the glycemic range (27, 28). In addition, the use of meals with unspecified carbohydrate content might have also influenced the meal misclassification observed in Howard et al. Indeed, we show smaller CVs for paired CGM comparisons for meals with greater carbohydrate content (>25 g). Sensor attachment sites might also explain reported differences (as referred to in the Methods). The concordance between devices for all glycemic metrics used in this study demonstrates the efficacy of CGMs in capturing different features of an individual's glycemic response to food and overall dietary patterns. This information is fundamental for the eventual implementation of personalized glycemic recommendations based on CGM measurements.

Some limitations of this study should be considered. Advanced error correction methods were not applied to the data intentionally to mimic the “real-life scenario” in which CGMs are used in personalized nutrition. However, future studies investigating CGM-generated data across different studies would benefit from applying error correction. CGM device allocation was not randomized and there were marked differences in the numbers of participants allocated to 2 of the same CGM brand or 2 different CGM brands. The groups differed in the number of female participants as well as other baseline characteristics; this trend was similar when comparing individuals monitored with 1 or 2 devices. Our data were collected using the FSL sensor and the DEX system, so our results may not be generalizable to other CGM systems; however, the performance of conventional CGM systems is largely similar (28). We were unable to replicate our results in an independent data set, but to the best of our knowledge, no studies of similar character are available for replication purposes. We did not investigate 1-h glucose responses or other postprandial glycemic parameters (e.g., peak concentrations) that may be relevant for meal ranking categorization. Although readings at different time points may provide valuable information on meal ranking concordance and CGM accuracy, we elected to use 2-h glucose iAUC because it has been used as a reference value for predicting postprandial glycemic responses (4) and is highly correlated with other postprandial glycemic parameters, while also maintaining a focused scope for this report. Although standardized meals were consumed either singularly or in duplicate, and the meal order was block randomized, it is possible that time differences may have biased our estimates for the ad libitum meals. Finally, the inclusion of healthy participants might limit the generalizability of our findings to other populations. However, previous studies conducted in individuals with diabetes have shown a good correlation between CGM devices and capillary blood (29–31) and high predictive accuracy in diabetes models (32). Nevertheless, further technological advances to improve interstitial glucose-sensing accuracy are needed, especially during the postprandial state in which rapid changes occur in glucose concentrations (within minutes) as well as alterations in blood flow rate or body temperature (27).

In conclusion, our data provide evidence to support the repeatability and concordance of CGM sensors in assessing glycemic responses through various glycemic metrics and meal ranking categorization. These data also support the hypothesis that observed variation in glycemic responses is influenced by within-subject variation and meal characteristics rather than the CGM device. Our results are critical for the continuing investigation of the determinants and variability of glycemic responses and the potential for the use of CGMs in personalized nutrition in the near future.

## Supplementary Material

nqac026_Supplemental_File

## Data Availability

Data described in the article, code book, and analytic code are held with the Department of Twin Research at King's College London and will be made available using our normal procedures overseen by the Wellcome Trust and its guidelines as part of our core funding. The application is at https://twinsuk.ac.uk/resources-for-researchers/access-our-data/.

## Notes

Supported by Zoe Ltd; TwinsUK is funded by the Wellcome Trust, Medical Research Council, Versus Arthritis, European Union Horizon 2020, Chronic Disease Research Foundation (CDRF), Zoe Global Ltd and the National Institute for Health Research (NIHR) Clinical Research Network (CRN) and Biomedical Research Centre based at Guy’s and St Thomas’ NHS Foundation Trust in partnership with King’s College London. SEB was supported in part by Biotechnology and Biological Sciences Research Council grant BB/NO12739/1. ATC was supported by a Stuart and Suzanne Steele MGH Research Scholar Award and R35 CA253185. PWF was supported in part by European Research Council grant CoG-2015_681742_NASCENT, the Swedish Research Council, the Novo Nordisk Foundation, and a Swedish Foundation for Strategic Research IRC award. AMV was supported by the National Institute for Health Research (NIHR) Nottingham Biomedical Research Centre. TwinsUK is funded by the Wellcome Trust, MRC, European Union, Chronic Disease Research Foundation, Zoe Ltd, and the NIHR-funded BioResource, Clinical Research Facility, and Biomedical Research Centre based at Guy's and St Thomas’ NHS Foundation Trust in partnership with King's College London. JM was supported by American Diabetes Association grant #7-21-JDFM-005 and NIH grant P30 DK40561.

Supplemental Tables 1–4, Supplemental Methods 1, and Supplemental Figures 1–3 are available from the “Supplementary data” link in the online posting of the article and from the same link in the online table of contents at https://academic.oup.com/ajcn/.

JM, IL, and KMB are joint first authors.

JM, IL, KMB, SEB, and AMV contributed equally to this work.

Abbreviations used: CGM, continuous glucose monitor; DEX, Dexcom G6 continuous glucose monitor; FSL, Abbott Freestyle Libre Pro continuous glucose monitor; glucose_iAUC0–2 h_, incremental area under the glucose curve between 0 and 2 h; GV, glycemic variability; OGTT, oral-glucose-tolerance test; PREDICT, Personalised Responses to Dietary Composition Trial; TIR_ADA_, time in range according to the American Diabetes Association cutoffs; TIR_ND_, time in range according to nondiabetic adjusted cutoffs.
